# Advancements in superconducting quantum computing

**DOI:** 10.1093/nsr/nwaf246

**Published:** 2025-06-17

**Authors:** Yao-Yao Jiang, Chunqing Deng, Heng Fan, Bing-Yang Li, Luyan Sun, Xin-Sheng Tan, Weiting Wang, Guang-Ming Xue, Fei Yan, Hai-Feng Yu, Ying-Shan Zhang, Yu-Ran Zhang, Chang-Ling Zou

**Affiliations:** Beijing Key Laboratory of Fault-Tolerant Quantum Computing, Beijing Academy of Quantum Information Sciences, Beijing 100193, China; Institute of Physics, Chinese Academy of Sciences, Beijing 100190, China; University of Chinese Academy of Sciences, Beijing 101408, China; Quantum Science Center of Guangdong-HongKong-Macao Greater Bay Area, Shenzhen 518045, China; Beijing Key Laboratory of Fault-Tolerant Quantum Computing, Beijing Academy of Quantum Information Sciences, Beijing 100193, China; Institute of Physics, Chinese Academy of Sciences, Beijing 100190, China; Center for Quantum Information, Institute for Interdisciplinary, Information Sciences, Tsinghua University, Beijing 100084, China; Beijing Key Laboratory of Fault-Tolerant Quantum Computing, Beijing Academy of Quantum Information Sciences, Beijing 100193, China; Institute of Physics, Chinese Academy of Sciences, Beijing 100190, China; University of Chinese Academy of Sciences, Beijing 101408, China; Center for Quantum Information, Institute for Interdisciplinary, Information Sciences, Tsinghua University, Beijing 100084, China; School of Physics, Nanjing University, Nanjing 210093, China; Center for Quantum Information, Institute for Interdisciplinary, Information Sciences, Tsinghua University, Beijing 100084, China; Centre for Quantum Technologies, National University of Singapore, Singapore 117543, Singapore; Center for Quantum Information, Institute for Interdisciplinary, Information Sciences, Tsinghua University, Beijing 100084, China; School of Physics, Nanjing University, Nanjing 210093, China; Beijing Key Laboratory of Fault-Tolerant Quantum Computing, Beijing Academy of Quantum Information Sciences, Beijing 100193, China; Center for Quantum Information, Institute for Interdisciplinary, Information Sciences, Tsinghua University, Beijing 100084, China; Beijing Key Laboratory of Fault-Tolerant Quantum Computing, Beijing Academy of Quantum Information Sciences, Beijing 100193, China; Beijing Key Laboratory of Fault-Tolerant Quantum Computing, Beijing Academy of Quantum Information Sciences, Beijing 100193, China; Center for Quantum Information, Institute for Interdisciplinary, Information Sciences, Tsinghua University, Beijing 100084, China; School of Physics and Optoelectronics, South China University of Technology, Guangzhou 510640, China; CAS Key Laboratory of Quantum Information, University of Science and Technology of China, Hefei 230026, China; Center for Quantum Information, Institute for Interdisciplinary, Information Sciences, Tsinghua University, Beijing 100084, China; Hefei National Laboratory, Hefei 230088, China

**Keywords:** superconducting quantum computing, superconducting qubit, quantum gate, quantum error correction, quantum simulation

## Abstract

Superconducting quantum computing (SQC) has achieved remarkable progress in recent years, garnering significant scientific and technological interests. This review provides a concise overview of the historical development of SQC, detailing fabrication methodologies for superconducting quantum chips and implementations of quantum gate operations. It compiles experimental progress in SQC over the past few years, including the preparation of multi-qubit entangled states, random circuit sampling experiments, demonstrations of quantum error correction based on surface codes, error mitigation techniques and quantum simulations. This review also discusses experimental progress related to boson-encoded qubits, fluxoniums and qudits. Finally, the current challenges in scaling are analyzed, and potential solutions for addressing these limitations are explored.

## Teaser

This review summarizes the development of superconducting quantum computing, including recent experimental breakthroughs, challenges in scalability, and the promising directions for future advancements.

## INTRODUCTION

Quantum computing represents a revolutionary computational paradigm that employs quantum processors—physical systems engineered to operate according to quantum mechanical principles—to perform information processing via the encoding, manipulating and measuring of quantum states. The unique quantum phenomena of entanglement and superposition enable quantum computers to offer significant advantages over classical counterparts for specific problem sets. Among the various hardware approaches to practical quantum computing, superconducting quantum computing (SQC) stands out as a promising method. This is largely due to its compatibility with existing semiconductor fabrication techniques and its potential for scalability, making it a key contender in developing more advanced quantum technologies. SQC can be categorized into two primary operational models: digital quantum computers, which execute quantum circuits through gate operations, and analog quantum computers, which simulate the evolution of Hamiltonians. Theoretical analyses have demonstrated the equivalence between these two methodologies. In terms of qubit encoding, SQC can utilize either two-level systems or bosonic modes, including cat codes [1], binomial codes [2] and Gottesman–Kitaev–Preskill (GKP) codes [3]. This review focuses primarily on the gate-based model of SQC. It provides a brief history of SQC, the fabrication of qubits, quantum gates, recent experimental advancements and developments in alternative qubit systems, concluding with a discussion of the near-term challenges in building large-scale SQC.

## BRIEF HISTORY OF SQC

From the 1980s to the 1990s, several macroscopic quantum phenomena were experimentally demonstrated in Josephson junctions and superconducting quantum interference devices (SQUIDs). These phenomena included energy-level quantization [4], macroscopic quantum tunneling [5], macroscopic resonant tunneling [6] and quantum superposition between two macroscopically distinct states [7]. These observations laid the foundation for the development of superconducting (SC) quantum computation.

In 1999, Nakamura *et al.* reported coherent oscillations between two charge states in a Josephson junction, marking the realization of the first SC qubit [8]. By tuning the ratio between the Josephson energy and the charging energy, various types of qubits were developed, including charge qubits [8,9], flux qubits [10] and phase qubits [11–13].

The theoretical framework of circuit quantum electrodynamics (cQED) [14], inspired by cavity QED principles, significantly advanced SC qubit measurement through quantum non-demolition readout techniques. A breakthrough occurred in 2007 with the proposal of the transmon qubit by Koch *et al.* [15,16]. Its simplified geometric configuration and reduced susceptibility to charge noise quickly made it the dominant superconducting architecture. In 2013, Barends *et al.* further optimized this design, introducing the Xmon variant [17]. This modification involved relocating the qubit from an internal resonator to external coupling, which enhanced spatial reconfigurability, enabled direct qubit-qubit coupling and facilitated scalable quantum chip integration using multiplexed readout resonators on a single transmission line.

In 2014, Barends *et al.* achieved single-qubit gate fidelities of 99.94% and two-qubit gate fidelities of 99.4% on a five-qubit sample, surpassing the threshold required for surface code error-correction schemes [18]. This milestone represented a significant advancement in SQC and marked the beginning of multi-qubit research. Historically, research efforts have prioritized extending qubit coherence times and reducing two-qubit gate errors, improving qubit readout fidelity, preparing multi-qubit entangled states, demonstrating quantum advantage, implementing quantum error correction (QEC) and conducting quantum simulations. These topics will be explored in more detail in the following sections.

## FABRICATION OF SC QUBITS

The fabrication process for SC quantum chips largely leverages techniques from the semiconductor industry. The workflow typically includes substrate cleaning, thin-film deposition, lithography, etching and packaging. A critical metric for evaluating the performance of a quantum chip is its coherence time. Several factors have been identified as detrimental to quantum coherence, including two-level systems (TLSs) in lossy materials [19], quasiparticles [20], magnetic vortices [21], parasitic modes [22] and spontaneous emission [23]. To achieve high-performance quantum chips, the fabrication process must be meticulously designed to meet performance requirements while minimizing impurities and defects in solid-state materials.

**Figure 1.**
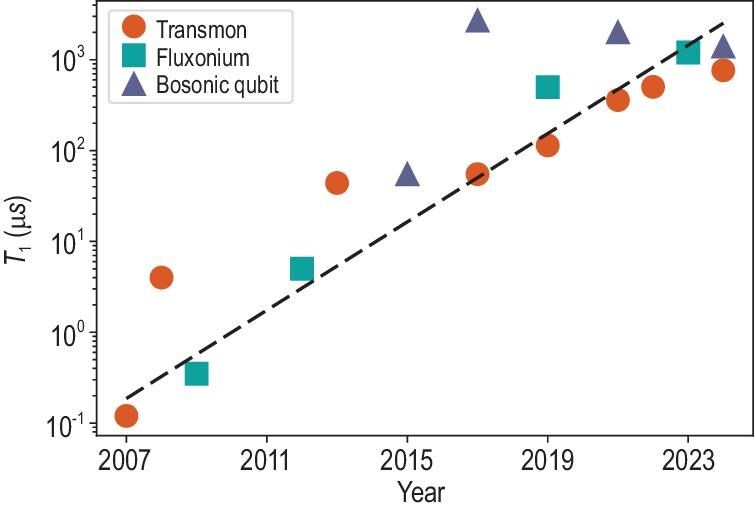
Energy relaxation time $T_1$ reported over the past years for SC qubits. The references are [17,22,24–29] for the transmon qubits, [30–33] for the fluxonium qubits and [34–37] for the bosonic qubits.

After over 20 years of effort, significant advancements have been made in enhancing the quantum coherence of SC qubits. Coherence times have dramatically increased from nanoseconds to milliseconds. As shown in Fig. 1, the logarithm of the SC qubit lifetime increases linearly with time.

### Chip architecture

The higher the number of qubits in a quantum chip, the more complicated the chip design. Single-layer wiring chips can barely meet the demand, and hence multi-layer wiring schemes must be developed. Flip-chip bonding and the through-silicon via (TSV) are combined in SC quantum chips to achieve multi-layer wiring. These processes enable multi-layer design schemes that separate qubits and wiring, facilitating more complex and scalable quantum circuits. In the flip-chip bonding process, indium (In) is exploited as a material for making superconducting connections and providing mechanical support. To be compatible with aluminum (Al) materials, a nitride material is also required as an under-bump metalization layer between the Al film and In to prevent the formation of Al-In alloys. In the TSV process, silicon vias are formed using the conventional Bosch process of deep silicon etching. The vias are filled with TiN by atomic layer deposition [38,39].

### Superconducting materials

The SC materials employed in the construction of quantum chips include both pure metals and compounds. Al is the most widely used SC material, with thin films deposited using various methods such as sputtering, electron-beam evaporation, thermal evaporation and molecular beam epitaxy. Another commonly used material is niobium (Nb), which boasts the highest critical transition temperature (9.2 K) and a high critical magnetic field (0.2 T) among pure metals, along with excellent mechanical properties. Nb thin films are typically deposited via sputtering. However, one drawback of Nb is its multiple valence states, leading to the formation of complex surface oxides when exposed to air. To mitigate losses due to these oxides, careful surface treatment is essential [40].

Recently, tantalum (Ta) has been identified as a material that can enhance coherence times [27,28]. Ta thin films are generally grown by sputtering. Because of their ability to form an equilibrium $\alpha$ phase (body-centered-cubic structure with a critical temperature of 4.5 K for $\alpha$-Ta) and a metastable $\beta$ phase (tetragonal structure with a critical temperature of 0.6–1 K for $\beta$-Ta), achieving stable $\alpha$-Ta films requires either high-temperature growth or the use of a seed layer.

The primary compounds used in making SC quantum chips are metal nitrides, including NbN (with a critical temperature of 16 K), TiN (6 K) and NbTiN (18 K). These thin films are formed through reactive sputtering. Their long London penetration depth results in significant kinetic inductance, introducing nonlinear characteristics into SC circuits. This nonlinearity limits their application in large-scale SC chips [41,42].

### Josephson junction

The Josephson junction (JJ) is a critical building block for SC quantum chips. A JJ consists of a superconductor–insulator–superconductor tri-layer structure. The most commonly used material for constructing JJs is Al, which has a superconducting transition temperature of 1.1 K. When exposed to oxygen, a thin layer of aluminum oxide (AlOx) with a thickness of $\sim\! 1$–2 nm forms on the surface of the Al metal. Since AlOx is insulating, it serves as an effective tunneling barrier. The Al/AlOx/Al JJ is typically fabricated using the shadow evaporation technique [43–45], which deposits the bottom and top Al layers at two different angles. After the growth of the bottom Al layer, the AlOx tunneling barrier is formed through thermal oxidation before the deposition of the top Al layer.

A second method involves a planar technique for overlap junctions, which does not require double-angle evaporation and is suitable for large-scale chip fabrication [46,47]. However, this method necessitates argon ion milling to remove the oxide layer of the Al film, potentially increasing surface roughness and introducing TLSs. Various processes have been developed to refine the fabrication of JJs. For instance, before depositing the bottom Al layer, oxygen plasma ashing can be employed to reduce organic residues on the substrate surface. The ‘bandage’ method eliminates the need for argon ion milling before Al evaporation, thereby reducing substrate damage caused by argon particles.

Thermal annealing of JJs is a key process for enhancing the performance and reliability of qubits by improving the crystallinity of the insulating barrier layer [48,49]. This enhancement reduces defects and impurities in the insulating oxide layer, thereby increasing coherence times and decreasing decoherence caused by TLSs in the amorphous dielectric. Additionally, thermal annealing can be used to adjust the resistance of the junctions, which is crucial for creating stable and reproducible tunnel barriers. This stability is essential for scalable frequency trimming in fixed-frequency transmon qubits. The annealing process also helps to reduce surface roughness and optimize the interface between layers, thus minimizing losses and improving the performance of SC circuits. In certain application scenarios, thermal annealing combined with other techniques, such as laser heating, provides precise localized rework capabilities [50].

## GATE OPERATIONS OF SC QUBITS

At the heart of quantum computation are quantum algorithms, which are executed through circuits composed of quantum gates that manipulate the states of qubits via unitary operations. In practical implementations, these circuits are typically constructed using combinations of single-qubit and two-qubit gates. For SC qubits, gates are realized through precise control of electromagnetic fields to manipulate their quantum states. High gate fidelity or a low gate error rate is the ultimate goal when constructing a gate scheme. Since decoherence and control errors are the two major sources of gate errors, gate speed and accuracy, which often contradict each other, have to be considered together and balanced. This section mainly focuses on the principles and progress of single-qubit and two-qubit gates.

### Single-qubit gates

Single-qubit gates perform operations on the quantum state of an individual qubit, allowing for rotations and phase shifts to be applied. An arbitrary single-qubit gate can be defined as


**Figure 2.**
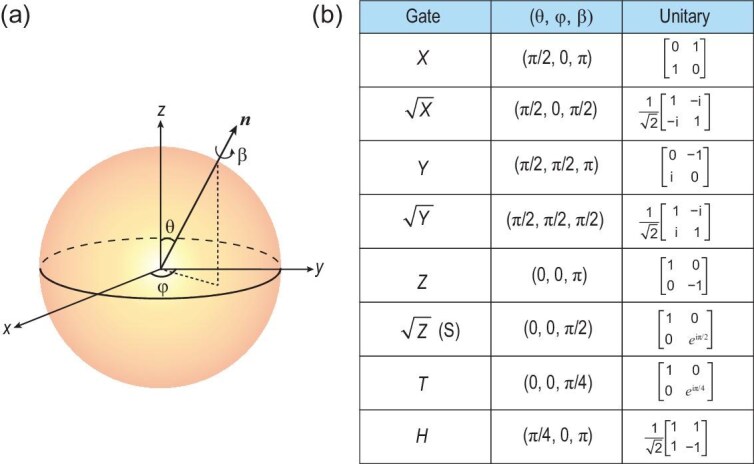
Bloch-sphere representation and common operations of single-qubit gates. (a) An arbitrary single-qubit gate as a rotation in the Bloch picture. (b) List of a few common single-qubit gate operations.

(1) \begin{eqnarray*}
R_n(\beta ) = e^{-\mathrm{i} \beta \boldsymbol{n} \cdot \boldsymbol{\sigma }/2},
\end{eqnarray*}

where $\boldsymbol{n}$ is a unit vector and $\boldsymbol{\sigma } = (\sigma _x, \sigma _y, \sigma _z)$ are the Pauli matrices. As illustrated in Fig. 2a, this operation corresponds to a right-handed rotation by an angle $\beta$ around the axis defined by $\boldsymbol{n} = (\sin \theta \cos \varphi , \sin \theta \sin \varphi , \cos \theta )$ in the Bloch sphere representation. Several common single-qubit gates are summarized in Fig. 2b.

In SC circuits, single-qubit gates are typically implemented using a microwave pulse resonant with the qubit’s transition frequency. The Hamiltonian of a qubit-shaped microwave pulse in the reference frame rotating at the frequency of an external drive can be expressed as


(2) \begin{eqnarray*}
H = \Delta \, \sigma _z/2 + \Omega ( \sigma _x\cos \phi + \sigma _y\sin \phi )/2 ,
\end{eqnarray*}

where $\Delta$ is the detuning between the qubit frequency and drive frequency, $\Omega$ is the drive amplitude or Rabi frequency and $\phi$ is the drive phase. A resonant pulse ($\Delta$ = 0) induces a rotation around an axis in the *X*-*Y* plane ($\theta = \pi /2$). The polar angle of the drive axis $\varphi$ is determined by the pulse phase, while the rotation angle $\beta$ is set by the time integral of the pulse amplitude (i.e. the Rabi frequency). For example, *X* and *Y* gates are both called $\pi$ gates as $\beta =\pi$, but with different drive phases, $\phi$ = 0 for *X* and $\phi =\pi /2$ for *Y*; their square root counterparts, $\sqrt{X}$ and $\sqrt{Y}$ gates, are called $\pi /2$ gates as $\beta =\pi /2$. For phase gates or *Z* rotations ($\theta = 0$), the virtual-*Z* technique is commonly employed. This approach updates the relative phases of subsequent pulses, effectively creating the *Z* rotation without requiring additional physical pulses. Arbitrary single-qubit rotations can be conveniently decomposed into two $\sqrt{X}$ gates interspersed with phase gates.

Achieving high-fidelity single-qubit gates requires careful calibration to account for hardware imperfections [51]. For instance, transmon qubits, which exhibit low anharmonicity, are susceptible to leakage into higher energy levels during single-qubit operations. The derivative removal by adiabatic gate (DRAG) technique effectively mitigates such leakage by optimizing the pulse shape, thereby reducing unwanted transitions to higher states [52,53].

Alternative methods for single-qubit manipulation also exist. Recent advances have included higher-order DRAG corrections to further enhance single-qubit gate performance [54–56]. For example, diabatic pulses are particularly effective for small-gap fluxonium qubits, which typically have $\mathinner {|{0}\rangle }$-$\mathinner {|{1}\rangle }$ transition frequencies of the order of 100 MHz. Another approach involves using periodic nanosecond single flux quantum (SFQ) pulses instead of a single microwave pulse for coherent qubit state manipulation [57–59]. These techniques, while less conventional, offer unique advantages in specific scenarios and qubit platforms.

### Two-qubit gates

Two-qubit gates are a crucial building block of gate-based quantum computation. In SC circuits, qubit-qubit interactions are typically mediated by direct coupling between qubits or, via a coupler, either capacitively or inductively. The gates are activated through tailored pulse control over a combination of internal or external system parameters. There are multiple approaches to categorizing and describing two-qubit gates.

First, two-qubit gates can be categorized based on the final unitary operation generated, regardless of the control scheme. According to the Cartan or KAK decomposition [84], any two-qubit unitary $U \in \mathrm{SU}(4)$ can be expressed as


(3) \begin{eqnarray*}
U = (K_1 \otimes K_2) U_\mathrm{w} (K_3 \otimes K_4),
\end{eqnarray*}

where


**Figure 3.**
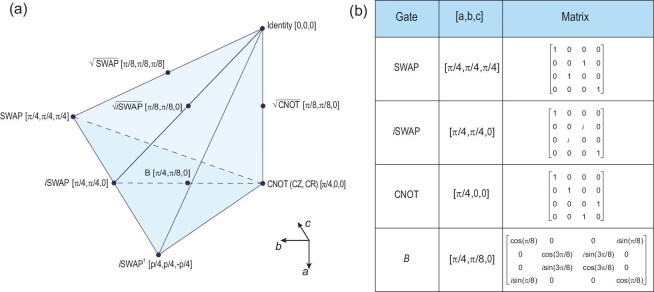
Weyl chamber and two-qubit gates. (a) Weyl chamber and common two-qubit gates marked with their coordinates. (b) List of selected two-qubit gate operations and corresponding unitary matrices.

(4) \begin{eqnarray*}
U_\mathrm{w}(a,b,c)= \exp [\mathrm{i} (a \sigma _x\sigma _x + b \sigma _y\sigma _y + c \sigma _z\sigma _z)],
\end{eqnarray*}

and $K_1, K_2, K_3, K_4 \in \mathrm{SU}(2)$ are single-qubit unitaries while $a, b, c \in \mathbb {R}$. Here $U_\mathrm{w}$ is unique for a given *U*. Because of periodicity and symmetries, all the possible $U_\mathrm{w}$ can be reduced to a tetrahedron called the Weyl chamber in three-dimensional (3D) Cartesian space spanned by the three coordinates $[a,b,c]$, as shown in Fig. 3. Unitaries sharing the same coordinates are said to be locally equivalent as they differ only by single-qubit operations. For example, the controlled-NOT (CNOT) gate, a fundamental gate in quantum algorithms, is located at $[\pi /4, 0, 0]$ in this framework. The commonly seen hardware-native gates, the controlled-*Z* (CZ) gate [71,85,86]


(5) \begin{eqnarray*}
U_{\mathit{CZ}} = \left[ \begin{array}{cccc}
1 &\quad 0 &\quad 0 &\quad 0 \\
0 &\quad 1 &\quad 0 &\quad 0 \\
0 &\quad 0 &\quad 1 &\quad 0 \\
0 &\quad 0 &\quad 0 &\quad -1 \end{array}\right]
\end{eqnarray*}

and the cross-resonance (CR) gate [65]


(6) \begin{eqnarray*}
U_{\text{CR}} =
\left[\begin{array}{cccc}
1 &\quad 0 &\quad -i &\quad 0 \\
0 &\quad 1 &\quad 0 &\quad i \\
-i &\quad 0 &\quad 1 &\quad 0 \\
0 &\quad i &\quad 0 &\quad 1
\end{array}\right],
\end{eqnarray*}

are the local equivalents of the CNOT gate.

From the control perspective, two-qubit gates can be categorized based on the system parameters that can be adjusted, generally including qubit energy levels, couplings and external drives. For instance, the system Hamiltonian for coupled transmon qubits is


(7) \begin{eqnarray*}
H &=& \sum _{i=1,2} \bigg ( \omega _i a_i^\dagger a_i + \frac{\alpha _i}{2} a_i^\dagger a_i^\dagger a_i a_i \bigg ) \\
&& +\, \frac{g}{2}(a_1^\dagger + a_1)(a_2^\dagger + a_2) \\
&& +\, \sum _{i=1,2} \Omega _{\mathrm{d}i} \cos (\omega _{\mathrm{d}i} t+\phi _{\mathrm{d}i}) (a_i^\dagger + a_i),
\end{eqnarray*}

where the $\omega _i$ are the qubit frequencies, the $\alpha _i$ are the anharmonicities, *g* is the coupling strength, and $\Omega _{\mathrm{d}i}$, $\omega _{\mathrm{d}i}$ and $\phi _{\mathrm{d}i}$ are the drive amplitudes, frequencies and phases, respectively.

The controllability of these parameters depends on the hardware architecture. In fixed-frequency fixed-coupling systems, external microwave drives are the sole control freedom. The CR gate, which activates a $ZX$-type interaction by driving one qubit at the frequency of another, is a prominent example. Another example is the microwave-activated phase gate, which leverages transitions to higher energy levels [87].

In systems with tunable qubits, two-qubit gates can be implemented by bringing specific energy levels into resonance. For instance, the *i*SWAP gate utilizes the $|01\rangle$-$|10\rangle$ transition, while the CZ gate uses the $|11\rangle$-$|20\rangle$ transition. The final effect of the CZ gate is to accumulate a non-trivial $\pi$ phase on the $\mathinner {|{11}\rangle }$ state. It can be realized through either adiabatic processes, which rely on slow frequency tuning, or adiabatic (non-adiabatic) processes, which enable faster gate operations—less decoherence errors—but become hard to calibrate and more sensitive to pulse distortion [71,72].

Tunable coupling architectures offer significant enhancements in gate fidelity and scalability. By dynamically modulating the coupling strength, these architectures circumvent the adiabatic limit, enabling fast and high-fidelity operations while minimizing crosstalk with neighboring qubits [88]. Although this approach requires additional control lines and meticulous calibration, it has demonstrated considerable success in achieving high-fidelity two-qubit gates in recent years. Note that combining fixed-frequency qubits with tunable couplers can simplify calibration and reduce the number of control lines while still allowing for the implementation of adiabatic or parametric gates [82,83,89].

Parametric gates, which involve modulating system parameters such as qubit or coupler frequencies at a specific frequency targeting a desired transition, provide another versatile control scheme. Examples include parametric *i*SWAP and CZ gates, where the qubit frequency is modulated targeting the $|01\rangle$-$|10\rangle$ and $|11\rangle$-$|20\rangle$ transitions, respectively [75,76,90]. Additionally, parametric drives can also be applied to the coupler to enable gates like *b*SWAP or *i*SWAP by driving at the sum or difference of the qubit frequencies [73].

Two-qubit gate schemes vary in qubit type, tunability, and control method, with key differences and trade-offs summarized in Table 1. The choice of an optimal two-qubit gate scheme depends on a range of factors, including gate performance, hardware constraints and the compilation strategies required for specific quantum algorithms [91]. Systems offering a broader variety of native gates are often preferred, as they provide greater flexibility in algorithm compilation, reduce gate counts and lower circuit depths, thereby improving overall performance. Continuous gate families such as the fSim [83] and $XY$ [92] families have been explored for their expressiveness and utility. More recently, a versatile gate scheme capable of generating arbitrary native two-qubit gates efficiently has been demonstrated, facilitating future hardware and algorithm co-design [83].

**Table 1.** Two-qubit gates realized with superconducting qubits. ‘Tr’, ‘Fl’ and ‘Res’ denote transmon, fluxonium and resonator, respectively; ‘ff’ denotes fixed frequency, while ‘tun’ denotes frequency tunable; ‘ad’, ‘di’ and ‘mi’ denote ‘adiabatic’, ‘diabatic’ and ‘microwave’, respectively.

$\Omega$	$\omega$	*g*	$g-\omega$
**Tr(ff)-Tr(ff):**	**Tr(tun)-Tr(tun):**	**Tr(ff)-Tr(tun)-Tr(ff):**	**Tr(tun)-Tr(tun)-Tr(tun):**
$\bullet$ mi-CZ [60]	$\bullet$ di-CZ [69]	$\bullet$ di-CZ [75]	$\bullet$ di-CZ [81]
$\bullet$ CR/CZ [61]	$\bullet$ di-CZ [70]	$\bullet$ ad-CZ [76]	$\bullet$ di-CZ/di-*i*SWAP [82]
$\bullet$ mi-CZ [62]	$\bullet$ ad-CZ [71]	$\bullet$ ad-CZ [77]	
	$\bullet$ di-CZ [72]		**Others:**
**Tr(ff)-Res-Tr(ff):**	$\bullet$ di-CZ/di-*i*SWAP [73]	**Others:**	$\bullet$ fSim [83]
$\bullet$ CR [63]		$\bullet$ CZ(RIP) [78]	
$\bullet$ CR [64]	**Fl(tun)-Fl(tun):**	$\bullet$ ad-CZ [79]	
$\bullet$ CR [65]	$\bullet$ *i*SWAP [74]	$\bullet$ ad-CZ [80]	
**Fl(tun)-Tr(tun)-Fl(tun):**			
$\bullet$ CZ [66]			
**Fl(ff)-Fl(ff):**			
$\bullet$ CR [67]			
**Others:**			
$\bullet$ *b*SWAP [68]			

The performance of two-qubit gates has steadily improved, with many research groups achieving gate errors below 1% and some recent results reaching errors under 0.1% [66,80], well below the surface code error threshold necessary for fault-tolerant quantum computing. However, achieving such high performance consistently across large-scale arrays of qubits remains a formidable challenge. Factors such as unwanted crosstalk between qubits, spurious defects, fabrication variability and other hardware imperfections introduce significant obstacles. Addressing these issues will be essential for scaling up quantum processors while maintaining high fidelity and uniform performance across all two-qubit gates.

## RECENT EXPERIMENTAL PROGRESS IN SQC

### Preparation of multi-qubit entangled states

Multi-qubit entanglement is central to the revolutionary potential of quantum computing. Unlike classical systems, entangled qubits exhibit non-local correlations that enable exponential parallelism, allowing quantum algorithms to process vast Hilbert spaces simultaneously. In the field of SQC, the preparation of multi-qubit entangled states remains a major research challenge. It requires ultra-precise quantum operations to synchronize multiple qubits into desired entangled states while minimizing decoherence and other environmental noise. Successfully achieving large-scale entanglement is not only important for advancing fundamental science, but also crucial for practical quantum computing applications. Over the past decade, the preparation of entangled states in SQC systems has mainly focused on Greenberger–Horne–Zeilinger (GHZ) states and cluster states [93]. Table 2 summarizes recent experiments on GHZ states in SQC.

**Table 2.** Summary of entangled GHZ states.

Entangled states	Fidelity	Group	Year
3-GHZ state [94]	0.88	Yale	2010
3-GHZ state [95]	0.62	UCSB	2010
10-GHZ state [96]	$0.668\pm 0.025$	ZJU	2017
18-GHZ state [97]	$0.525\pm 0.005$	ZJU	2019
18-GHZ state [98]	$0.5165\pm 0.0036$	IBM	2020
27-GHZ state [99]	$0.546\pm 0.017$	Melbourne	2021
29-GHZ state [100]	$0.506\pm 0.008$	IBM	2022
32-GHZ state [101]	$0.519\pm 0.014$	IBM	2024
60-GHZ state [102]	$0.595\pm 0.008$	ZJU	2024

### Demonstration of quantum advantages

The question of whether quantum computers can surpass the capabilities of state-of-the-art classical computers remains a central challenge in quantum computing. Quantum random circuit sampling (RCS) has become a key experimental framework for tackling this challenge. In RCS, random quantum gate operations are applied to multi-qubit systems, generating ensembles of quantum states whose probability distributions are then sampled. Classical simulation of these processes becomes intractable as circuit depth and qubit count increase, due to the exponential scaling of quantum state space dimensionality. While RCS currently has no direct practical applications, it serves as an important benchmark for assessing quantum computational advantage. Since 2019, six landmark RCS experiments have been conducted by research groups including Google and USTC, as summarized in Table 3.

**Table 3.** Summary of different RCS experiments, where XEB (cross-entropy benchmarking) fidelity serves as a metric to evaluate the agreement between experimental and ideal output distributions.

Group	Qubit number and circuit depth	Error rate of the simultaneous two-qubit gate	Readout fidelity	XEB fidelity	Year
Google [103]	53, 20	6.2$\times 10^{-3}$	96.2$\times 10^{-2}$	2.2$\times 10^{-3}$	2019
USTC [104]	56, 20	7.6$\times 10^{-3}$	95.23$\times 10^{-2}$	6.6$\times 10^{-4}$	2021
USTC [105]	60, 24	6$\times 10^{-3}$	95.49$\times 10^{-2}$	3.7$\times 10^{-4}$	2022
Google [106]	70, 24	6.63$\times 10^{-3}$	98.92$\times 10^{-2}$	1.7$\times 10^{-3}$	2024
Google [106]	67, 32	3.5$\times 10^{-3}$	98.7$\times 10^{-2}$	1.5$\times 10^{-3}$	2024
USTC [107]	83, 32	3.75$\times 10^{-3}$	99.133$\times 10^{-2}$	2.5$\times 10^{-4}$	2024

### Quantum error correction

SC qubits are highly fragile and vulnerable to environmental interference, leading to errors. The error rate of the best existing two-qubit gates remains at least 10 orders of magnitude higher than what is needed for practical applications. As a result, reducing error rates through QEC techniques has become a primary focus in SQC. Classical computers already employ well-established error-correction methods, such as encoding information with redundant bits and using majority voting rules for error detection and correction. Quantum computers can draw inspiration from these techniques, but they face unique challenges. For example, the no-cloning theorem prevents the perfect replication of quantum states for error protection, while the collapse of measurement makes real-time observation of encoded qubits impractical. Additionally, qubits are subject to continuously varying phase errors.

To address these challenges, several QEC schemes have been proposed for SQC. Among these, the surface code has attracted significant attention due to its two-dimensional layout and its requirement for only nearest-neighbor interactions, which aligns well with the planar design of SC qubits [41,108]. This stabilizer code introduces redundant auxiliary qubits that are entangled with data qubits. Errors are detected through a series of projective stabilizer measurements, followed by syndrome analysis and decoding to correct them. According to the quantum threshold theorem, if the physical qubit error rate falls below a certain threshold, adding enough redundant qubits can reduce the logical error rate to acceptable levels [109]. The surface code boasts a relatively high quantum gate fidelity threshold of $\sim \!99.3\%$, and the current manipulation accuracy of SC qubits has surpassed this threshold. This progress has led to the widespread adoption and active exploration of surface codes in QEC research.

Table 4 summarizes the outcomes of surface code error-correction experiments in current SQC research. The notation $[n,k,d]$ represents an encoding scheme, where *n* physical qubits encode *k* logical qubits, and *d* indicates the code distance. Recent results from Acharya *et al.* [110] show that the logical qubit error rate has now fallen below that of physical qubits, marking the critical break-even point–a significant milestone in QEC.

**Table 4.** Summary of recent surface code experiments

${[}n,k,d]$	Group	Logical error rate	Year
[4, 1, 2]	ETH Zurich	$2.60 \pm 1.3\%$	2020 [116]
[9, 1, 3]	ETH Zurich	$3.20 \pm 0.10\%$	2022 [117]
[9, 1, 3]	USTC	$\sim \!3\%$	2022 [118]
[9, 1, 3]	Delft	4.73%	2024 [119]
[9, 1, 3]	IBM	3.70%	2023 [120]
[9, 1, 3]	Google	$3.028 \pm 0.023\%$	2023 [121]
[9, 1, 3]	Google	$0.580\pm 0.002\%$	2024 [122]
[9, 1, 3]	ZJU	${\sim}1\%{-}2.5\%$	2025 [123]
[25, 1, 5]	Google	$2.914 \pm 0.01\%$	2023 [121]
[25, 1, 5]	Google	$0.270 \pm 0.003\%$	2024 [122]
[49, 1, 7]	Google	$0.143 \pm 0.003\%$	2024 [122]

Despite these advances, surface codes have limitations. A major issue is the large number of redundant qubits they require, with the number of physical qubits scaling quadratically with the code distance, resulting in low coding density. To address this, researchers have adapted concepts from classical low-density parity-check (LDPC) codes, proposing quantum LDPC (qLDPC) schemes. These schemes aim to reduce the number of redundant qubits by increasing connectivity between qubits. For example, using qLDPC encoding, 288 highly connected qubits can achieve the same performance as 3000 nearest-neighbor coupled qubits in surface codes [111]. Another limitation is the Eastin–Knill no-go theorem, which restricts the implementation of certain encoded gates transversely [112]. To overcome this, methods such as magic-state distillation and lattice surgery have been developed [113–115].

### Error mitigation

In 2023, the IBM team implemented an error mitigation (EM) technique to demonstrate the simulation of the two-dimensional Ising model on a 127-qubit chip, with a circuit depth reaching up to 60 layers [124]. This scale of quantum circuits has exceeded the capabilities of classical brute-force simulations, highlighting the potential practical value of noisy intermediate-scale quantum (NISQ) computers, even in the absence of fully fledged QEC techniques. Although subsequent studies have shown that classical computers can effectively simulate certain aspects of these quantum circuits [125], we will not elaborate on those details here. Instead, our focus remains on the EM techniques.

One of the most widely used EM techniques is zero-noise extrapolation (ZNE), which works by amplifying the noise levels during circuit execution and then extrapolating the results back to the zero-noise limit. It has been successfully applied to enhance the accuracy of variational quantum algorithms and quantum simulations. Recent advancements in ZNE primarily focus on optimizing noise scaling strategies and incorporating machine learning to achieve more accurate extrapolation. In 2023, the IBM research team extended ZNE to larger quantum circuits, involving up to 26 qubits, 60 layers and 1080 CNOT gates [126], demonstrating ZNE’s potential for enabling classically intractable quantum simulations on NISQ devices. ZNE was also systematically validated on small QEC codes, using a 17-qubit rotated surface code (distance-3). Researchers reduced the logical qubit bias $\delta$—defined as the absolute deviation of the logical operator expectation value from its ideal value—from $\sim\! 5 \times 10^{-2}$ to $2 \times 10^{-2}$ after a single round of syndrome measurements, with consistent improvements across various logical states [123].

Probabilistic error cancelation (PEC) [127] is a sophisticated approach that aims to counteract the detrimental effects of noise in quantum systems by constructing an inverse map of the noise process. This method has shown great promise in enhancing the accuracy of quantum operations. However, its implementation is contingent upon a comprehensive understanding of the noise characteristics, often achieved through techniques like gate set tomography. PEC also involves sampling from error-corrected circuits to reconstruct the ideal outcomes, which adds complexity to the overall process.

Symmetry-based EM leverages conserved quantities in quantum circuits to identify and correct errors. For instance, enforcing particle number conservation in variational quantum eigensolvers has demonstrated significant improvements in algorithmic accuracy. Recent experiments on superconducting qubits have successfully applied symmetry-based techniques to enhance computational reliability.

Dynamic decoupling, which uses carefully designed pulse sequences to average out environmental noise, remains an effective method for mitigating decoherence. Adaptive pulse sequences that optimize noise suppression in real time have shown promise for extending coherence times and improving gate fidelities.

EM techniques are often combined to achieve superior results. For instance, integrating ZNE with symmetry-based methods or PEC has been proven to enhance computational reliability. These combined strategies serve as a crucial bridge between the current capabilities of quantum hardware and the requirements of fault-tolerant quantum computing. They enable significant progress in algorithm development and practical applications on NISQ devices.

### Quantum simulation

#### Simulating physical phenomena in atomic physics and quantum optics

As an artificial atom, the SC qubit offers excellent parameter controllability, making it an ideal platform for exploring various physical phenomena. Given that its dynamic behavior is often described using the terminology of quantum optics and atomic physics, SC circuits naturally lend themselves to demonstrating phenomena observed in optical and atomic systems. One notable example is the stimulated Raman adiabatic passage (STIRAP), a widely used state transfer protocol in optics known for its robustness. In the original STIRAP protocol, two pulses, labeled as pump and Stokes, are used to facilitate state transfer between $\Lambda$-type energy levels. In superconducting systems, similar Hamiltonians can be achieved with microwave pulses [128]. Recent research has further enhanced adiabatic evolution, incorporating superadiabatic and other shortcut-to-adiabaticity schemes [129,130]. This development elevates STIRAP from merely a demonstrative technique to a practical protocol for quantum state manipulation. Likewise, the Autler–Townes effect has been extensively studied in superconducting systems [131,132], with applications like measuring the band structure of simulated condensed matter materials [133]. Another phenomenon is electromagnetic induced transparency, a quantum optical phenomenon that makes materials transparent to a specific light frequency [134–136]. This feature is valuable for data storage in quantum information and computation.

Additionally, the parameter controllability of SC circuits enables the simulation of more complex quantum systems, even those involving only two energy levels. For instance, topological materials, whose electrical properties are protected by topological invariants, exhibit robustness to external disturbances. However, the parameters of natural or artificially synthesized materials cannot usually be freely adjusted to explore their physical properties in detail. By mapping momentum to the parameter space of an SC circuit, topological and geometric invariants, such as the Chern number and Berry phase, can be measured through carefully designed quantum evolutions. These measurements can characterize topological phase transitions [137]. Additionally, certain geometric and topological quantities, predicted by theoretical models, can be experimentally observed in designed experimental protocols. Examples include the topological properties of the Maxwell semimetal and the quantum metric tensor of the tensor monopole [138], while these topological phases have not yet been realized in physical materials.

Superconducting circuits also provide a systematic approach to studying non-Hermitian quantum systems. In typical superconducting qubit systems, unitary operations alone do not reveal the properties of open systems with gain and dissipation. Therefore, a quantum system with higher-dimensional parameters is needed to construct an equivalent non-Hermitian Hamiltonian within a subspace [139]. Through analysis of dynamic evolution data, a range of non-Hermitian phenomena, such as parity-time symmetry breaking and the geometric properties of exceptional points, can be demonstrated [140,141].

#### Quantum simulation of quantum many-body physics

The key idea of quantum simulation is to emulate relevant quantum models with a device that obeys the laws of quantum mechanics. While fault-tolerant devices for quantum computation require further development, a practical quantum advantage already exists in the quantum simulation of quantum many-body physics (QMBP) [142] in NISQ devices. In particular, to simulate dynamics of quantum many-body systems with a scalable analogue, quantum simulators are beyond the capability of classical supercomputers, but require fewer resources for an SC processor [143]. Combining more programmability with analog simulators, a hybrid digital-analog approach merges analog unitary blocks and digital quantum gates and preserves both the scalability and versatility of the analog and digital simulators, respectively [144]. Furthermore, digital quantum simulation, compatible with error correction, is applicable for more complex models with higher accuracy and broader programmability, notwithstanding the hardware requirements and computation time [145].

The implementation of quantum simulators using SC qubits has been attracting growing attention due to flexible designs of microchip fabrication, universal controllability and high-fidelity readouts. The circuit excitations, rather than physical particles subject to conservation laws, make SC processors an ideal platform for accessing emergent out-of-equilibrium physics. The non-equilibrium dynamics of an analog simulator can be seen as quantum walks (QWs) of all initially prepared excitations with time-dependent control. QWs of strongly interacting microwave photons were demonstrated on SC processors on a 12-qubit chain [146], a 24-qubit ladder [147], a 2D array of 62 qubits [148] and a 24-qubit ring [149]. Under a linear potential, QWs of a photon, representing the Bloch oscillation and Wannier–Stark localization, were probed on five-qubit [150] and nine-qubit [151] processors.

From another perspective, the dynamics of an analog quantum simulator behaves as an entangling operation on an initial state. The quench dynamics of a generic isolated quantum system tends to explore almost the entire configuration space with an exponentially growing number of quantum states in the system size, known as the eigenstate thermalization hypothesis (ETH) [152], which is intractable for a classical computer [153]. Quantum thermalization was demonstrated on SC processors by simulating the ergodic dynamics of a 12-qubit chain [154] and a 62-qubit system [155]. This is also related to quantum information scrambling, which can be characterized by the out-of-time correlators (OTOCs) and tripartite mutual information (TMI) [156]. The key challenge to measure OTOCs is to reverse the time evolution of the system [157]. By engineering quantum circuits on a 53-qubit processor, dynamics and fluctuation of OTOCs were probed to investigate scrambling of quantum information [158]. Qutrit information scrambling was demonstrated on a five-qutrit SC circuit [159]. With a digital-analog approach, OTOCs were probed for identifying quantum thermalization on a ($3\times 3$)-qubit processor [160]. Floquet engineering was applied to realize the reverse time evolution of the system and to investigate operator spreading in a 10-qubit chain [161]. On a 24-qubit ladder processor, TMI was measured to signal thermalization and information scrambling [162].

Benefiting from architectures with tunable interactions, high controllability and readout techniques, SC processors are also versatile for demonstrating various mechanisms of weak and strong ETH violations, including quantum many-body scars (QMBSs) [163], prethermalization [164], many-body localization (MBL) [165] and discrete time crystals (DTCs) [166]. A QMBS was realized, utilizing a 30-qubit SC processor [167]. Digital-analog quantum simulation of a prethermal phase was demonstrated on a 12-qubit SC processor [168]. An observation of prethermal topologically ordered time crystals with $3\times 8$ SC qubits was reported [169]. The MBL dynamics of a long-range interacting spin-$\frac{1}{2}$  $XY$ model was emulated by programming disorder and long-range interactions on a 10-qubit SC processor [170]. With linearly varied on-site potentials, Stark MBL was emulated on a 32-qubit processor [171]. By realizing the dynamics of a 12-qubit processor, MBL transitions [172,173] and the proximity effect in the overlap between localized and delocalized states [174] were investigated. The MBL transition in a 2D system was identified from a Fock-space perspective using $4\times 6$ SC qubits [175]. An energy-resolved MBL transition was simulated by controlling both disorder strength and initial-state preparation using 19 SC qubits [176]. With tunable controlled-phase gates on an array of SC qubits, an MBL DTC was realized [177].

A dynamic spectroscopy technique from the response of the system given local perturbations is compatible with SC simulators to measure energy levels of quantum many-body systems. This technique was applied to signal the thermalization-localization transition [178], to measure the topological band structure of Chern insulators [125] and to demonstrate the Hofstadter butterfly energy spectrum [178,179]. Therefore, SC simulators are capable of demonstrating the bulk-edge correspondence in topological phases of matter, when combining the measured topological band structure [125,179] and the observation of dynamical localization of edge excitations [125,179,180]. As a lower-dimensional topological pump shares the same topological origin as higher-dimensional topological physics, quantum pumps were realized on a 1D array of SC qubits [125,181,182], demonstrating 2D integer quantum Hall effects. In addition, two types of second-order topological pumps were demonstrated on a ($4\times 4$)-qubit processor [183]. Moreover, SC circuits are versatile for programming topological states that have never been prepared in real materials before, such as topologically ordered states [184], anyons with anyonic braiding [185–190], a lattice version of photonic fractional quantum Hall states [191] and Floquet symmetry-protected topological phases [192,193]. By applying the fast mid-cycle qubit readout technique for QEC, SC simulating platforms can also realize measurement-induced entanglement transitions [194].

Overall, with universal control and high-fidelity qubit readout, distinct designs of SC circuits perform an ideal platform for studying various emergent QMBP, including spin models [195–200], bosonic/fermionic models [201–203], black holes [204], multipartite entanglement detection [205,206], quantum chemistry [207–212] based on variational quantum algorithms [213], and quantum biology [214]. With further hardware development, fault-tolerant SC quantum processors that are compatible with QEC or quantum EM will access more complex QMBP with higher accuracy.

## OTHER QUBITS

### Bosonic qubit

**Figure 4.**
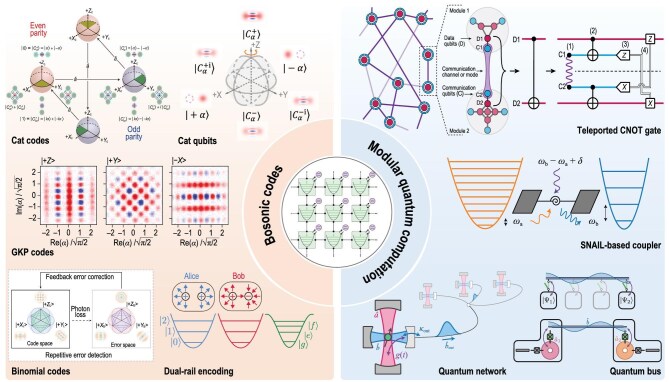
Advancements in cavity-based superconducting qubits. Cavity-based superconducting qubits are propelling the field of quantum information science forward with their applications in QEC and modular quantum computation. A limited set of examples is highlighted in this illustration. For QEC, cavity-based qubits are utilized in the development of bosonic codes, which enhance the fault tolerance of quantum systems. Reprinted with permission from [219–223]. Additionally, cavity-based qubits are integral to modular quantum computation, facilitating the execution of teleported CNOT gates and enabling the use of SNAIL- (superconducting non-linear asymmetric inductive element) based couplers to establish robust quantum networks. Reprinted with permission from [224–227].

Cavity-based superconducting qubits offer a promising alternative for encoding and manipulating quantum information, with the assistance of auxiliary qubits (e.g. transmons) providing nonlinear control and readout. Superconducting cavities act as quantum memories with significantly extended coherence times, owing to their effective isolation from environmental noise, a critical factor that constrains reliable quantum operations. The strong coupling between cavity modes and auxiliary qubits enables robust quantum gates and facilitates entanglement generation across multiple cavities, thereby supporting the development of scalable quantum systems [35,215–218]. This design takes advantage of the inherent properties of bosonic modes, including their large Hilbert space, long coherence time and low-loss propagation over long distances. Consequently, cavity-based superconducting qubits offer a unique quantum platform for realizing logical qubits protected by QEC and are compatible with other bosonic carriers, including magnons, phonons and optical photons. Advancements in cavity-based superconducting qubits are illustrated in Fig. 4.

From the perspective of universal quantum computation, cavity modes provide a hardware-efficient approach for introducing redundancy in novel quantum information encoding [222,228–230]. Recently, significant milestones have been achieved in demonstrating the break-even point of QEC using various codes, such as cat codes [1,231], binomial codes [2,219] and GKP codes [3,232]. These advancements represent a crucial step toward universal quantum computation, as they enhance the coherence time of logical qubits compared to those of cavity modes and ancillary qubits [233]. Additionally, fault-tolerant quantum gates have been successfully demonstrated for these cavity-based logical qubits [223,234,235]. In 2024, the realization of entangled logical qubits within this platform marked a significant advancement in the field [236].

Alternative strategies such as cat qubits [222,224–226,237,238] and dual-rail encoding [227,239–241] have also recently been introduced, each offering unique advantages for achieving fault tolerance. Cat qubits utilize continuous drives to define the system eigenstates as superpositions of coherent states, demonstrating significantly improved performance with biased noise, where bit-flip errors are greatly suppressed. This greatly lowers fault-tolerance thresholds and enhances resilience to environmental noise. Dual-rail encoding achieves redundancy by distributing a logical qubit across two separate cavities. This approach effectively mitigates photon loss errors through immediate error detection and correction, making it a robust choice for reliable quantum information storage and processing, particularly in larger systems. Furthermore, recent advances in hybrid quantum architectures that combine continuous-variable and discrete-variable encoding are broadening the scope of error correction and contributing to the development of more robust fault-tolerant designs [226,230,238].

In addition to the continued development of novel QEC codes and precise control technologies to go far beyond the break-even point, exploring distributed quantum computation schemes for scaling up the system is also of great importance [242,243]. Two promising approaches for scaling are (i) incorporating multiple cavities within a single module for each quantum node and (ii) connecting multiple quantum nodes to form a quantum network. For the first approach, recent research has focused on refining control precision for interactions across multiple cavities, a critical step in expanding cavity-based qubits into complex, scalable quantum processors [217,218,220,221,244–247]. The second approach involves exploring modular architectures by connecting small, precisely controlled units of cavity-mode qubits via transmission lines or waveguides. This enables coherent interaction and efficient state transfer between quantum modules. The modularity introduced by this design provides flexibility in system expansion, facilitating the development of larger quantum processors while maintaining high coherence and precision [248,249].

Looking ahead, the cavity-based qubits can be further extended in several ways. First, quantum networks and distributed quantum computation over long distances can be realized through optical interconnects, which link separate modules using light [229,230,250–252]. As bosonic modes, optical signals are naturally compatible with cavity-based qubits, providing an effective medium for quantum state transfer and entanglement generation across modules. Second, the number of qubits within a single chip can be significantly increased by incorporating phononic cavities. Phononic modes can couple linearly with superconducting qubits via piezoelectrical effects, and their mode density is significantly higher than that of electromagnetic modes due to the micron-sized phonon wavelength. This direction has garnered considerable interests [253–258]. Researchers have already demonstrated strong phonon-qubit coupling and reported phonon mode lifetimes comparable to those of on-chip superconducting cavities [256]. Finally, by leveraging advanced real-time feedback control techniques, the performances of cavity-based qubits can be further improved. These advancements not only benefit quantum computation, but also hold potential applications in other quantum fields, such as quantum metrology. [254,255,257,259].

### Fluxonium

**Figure 5.**
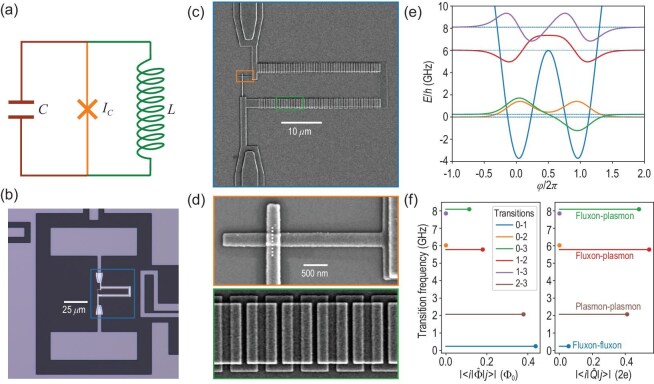
Full-stack architecture of a fluxonium. (a) Schematic of the fluxonium circuit, consisting of a Josephson junction (critical current $I_c$), a loop inductor (*L*) and a shunt capacitor (*C*). (b) Optical micrograph of a typical fluxonium qubit device. (c) Scanning electron microscopy (SEM) image of the qubit loop structure, highlighting the Josephson junction and junction array (blue box in (b)). (d) SEM images of the Josephson junction (orange box) and the junction array (green box). (e) Qubit eigenstate wave functions and energies are on a phase basis. (f) Transition matrix elements of the flux ($\Phi$) and charge (*Q*) operators.

Unlike charge qubits or transmons, which encode states in single Cooper-pair charge states or plasmon oscillations, flux qubits [7] encode quantum states in the flux across an SC circuit. The simplest flux qubit consists of an SC loop interrupted by a Josephson junction with loop inductance *L* (Fig. 5a). Its Hamiltonian resembles a point mass in a double-well potential, where fluxon states—localized in the potential minima—define the computational basis. A fluxonium [30] is a flux qubit operating in the regime $E_J>E_C>E_L$, where $E_J$, $E_C$ and $E_L$ are the Josephson, charging and inductive energies, respectively. Achieving this parameter regime requires a very high loop inductance *L*, necessitating a high-impedance superinductor with inductance in the hundreds of nanohenrys and impedance much larger than the vacuum impedance. Such superinductors are realized using arrays of JJs.

At the commonly used half-integer flux quantum bias, the fluxon states degenerate, creating a first-order flux-noise-insensitive point. Its single-island design makes the fluxonium immune to charge offset noise, similar to transmons and other flux qubits. The qubit states are symmetric and anti-symmetric superpositions of the two fluxon states, with an energy gap determined by the tunneling amplitude $\propto \exp (-8E_J/E_C)$. This results in an energy gap in the hundreds-of-megahertz-range order of magnitude lower than transmons, while higher excited states, corresponding to plasma oscillations, remain in the gigahertz range, similar to transmons, as shown in Fig. 5e. Relaxation is thus mitigated as its charge dipole scales quadratically with the qubit frequency, and its magnetic dipole is suppressed by the superinductor, reducing sensitivity to dielectric and inductive losses. These properties have enabled fluxonium qubits with coherence times exceeding 1 ms under laboratory conditions with moderate dielectric loss and flux noise [33,260].

This rich energy structure (Fig. 5f) enables high anharmonicity and versatile control schemes for high-fidelity quantum operations. Qubit readout is performed via dispersive coupling to a microwave resonator in a circuit QED setup. A strong dispersive shift, enabled by the 0-3 transition despite the large detuning ($> \!6$ GHz) between qubit and resonator frequencies, allows high-fidelity readout using homodyne detection [74]. The qubit’s low energy gap necessitates active initialization, achieved through quantum non-demolition readout with heralding [66] or sideband cooling [74]. By breaking selection rules with a flux offset, efficient sideband cooling enables the simultaneous reset of multiple qubits via frequency multiplexing [261]. Single-qubit gates are implemented by resonant driving, leveraging the qubit’s charge or magnetic dipole. These gates achieve fidelities exceeding 99.99% without advanced pulse shaping, as leakage is negligible [33].

Two-qubit gates exploit magnetic or charge dipole interactions. Inductive coupling, inherent to flux qubits, enables $ZZ$ [262], *i*SWAP [263] and cross-resonant interactions [264] with gate errors close to or below 0.001. However, achieving strong coupling is challenging due to the fluxonium’s large self-inductance. Current implementations use galvanically connected Josephson junction arrays to enhance mutual inductance, linking superconducting loops, but introducing challenges like flux crosstalk and ground loops in multi-qubit setups.

A promising approach for scaling fluxonium systems involves using charge coupling mediated by tunable transmon couplers [66,265]. Two-qubit gates are implemented via microwave-activated phase gates, utilizing transitions to non-computational levels. This method offers two key advantages over transmon systems. First, the weak direct qubit-qubit coupling, due to the small charge dipole of low-frequency fluxonium qubits, minimizes crosstalk and frequency crowding. Second, the interaction is tunable through the coupler and requires microwave activation, ensuring a high on-off ratio for qubit operations. Combined with frequency multiplexing, this enables parallel execution of many two-qubit operations with minimal microwave control resources.

The fluxonium is a more complex superconducting circuit than the transmon, designed to offer superior noise protection and a highly anharmonic energy spectrum. Its construction, requiring both a superinductor and a phase-slip junction, demands advanced engineering and fabrication techniques. High precision in the Josephson junction critical current is essential for accurate qubit frequencies, particularly for large-scale integration. However, the dielectric loss in fluxonium qubits, around $10^{-6}$, is nearly an order of magnitude worse than that of state-of-the-art transmons. Future advancements, including industry-standard fabrication and leveraging semiconductor manufacturing facilities [47], aim to enhance yield, parameter control and coherence times, potentially exceeding the millisecond level for very large-scale quantum processors.

### Qudit

The qudit, a *d*-level quantum system, utilizes multiple energy levels to encode information. The computational subspace of qudits has size $d^N$, where *N* is the qudit number. Given the complexity of further increasing *N* in hardware, we can instead increase *d*. With improved coherence and multi-tone control and measurement techniques, we are now motivated to explore superconducting qudits.

A superconducting phase qudit was first manipulated with up to $d=5$ [266]. But, by far, high-coherence transmons are the most popular superconducting qudit type [267]. High-fidelity control and measurements of a single qudit were implemented for $d=4$ [268], 8 [269] and 10 [270]. Two-qutrit gates were implemented on designs with fixed [159], tunable [271] and parametric coupling [272]. Tricks for qubits, such as echo [273], dynamical decoupling [274] and benchmarking methods [275], were generalized to qudits. A universal gate set based on CR was also proposed [276]. Qudit error correction is thriving, and GKP bosonic qudits were demonstrated with beyond break-even error-corrected performance [277]. Combining transmon and bosonic qudits in a hybrid system was also proposed [278].

Overall, qudits provide flexible control and efficient encoding, which indicates a scaling advantage over qubits [279]. However, scaling up with qudits remains challenging.

## NEAR-TERM CHALLENGES

To tackle real-world problems effectively, a quantum processor will ultimately need to incorporate millions of qubits. Achieving such integration will necessitate significant engineering efforts and innovations. Given the unpredictable nature of advancements in science and technology, it is challenging to outline a precise road map for developing quantum chips with millions of qubits. Instead, this section focuses on the key challenges that the SQC community must address to integrate hundreds of qubits on a single quantum chip. These challenges include improving qubit coherence times, minimizing crosstalk and noise, optimizing fabrication processes and enhancing coupling strengths. Addressing these issues will pave the way for scaling up to larger systems and achieving the practical quantum computing capabilities required for complex problem-solving.

### Quantum chip

Quantum chips are the cornerstone of superconducting quantum computers. An ideal quantum chip should exhibit several key characteristics: all qubits must be functional, their frequencies must align with design specifications, qubits should demonstrate long coherence times, the density of TLS defects should be minimal, coupling strengths should be optimized, crosstalk should be reduced and there should be rapid reset and readout capabilities.

Achieving these goals simultaneously is highly challenging. The first two criteria—functional qubits and precise frequency matching—are fundamental prerequisites for ensuring a quantum chip’s operational integrity. Achieving these requires stable fabrication processes, equipment that guarantees consistent processes and operation within a high-quality cleanroom environment. Coherence times and TLS defect densities are closely tied to the materials used and the fabrication process for qubits. While individual fixed-frequency qubit relaxation times have reached milliseconds, the best average relaxation time for frequency-tunable multi-qubit systems remains around 0.1 ms. To achieve a two-qubit gate error rate of $10^{-4}$ with an estimated gate length of 50 ns, qubit coherence times must exceed 0.5 ms. Given that the overall performance of the chip is often determined by its weakest component, significant enhancements in the coherence times of scaled qubits remain crucial.

TLS defects pose a critical challenge for SC quantum chips. Their unpredictable frequency distribution and temporal variability interfere with qubit manipulation, necessitating frequent recalibration. Addressing this issue involves optimizing the materials and fabrication processes used in qubit preparation.

The final three factors—appropriate coupling strengths, minimized crosstalk and fast reset/readout capabilities—are design related and require precise electromagnetic simulations and iterative experimental testing to identify suitable configurations. Achieving these design objectives demands rigorous validation through experimentation to ensure that the final quantum chip meets the stringent requirements for practical quantum computing applications. This meticulous approach ensures not only functionality, but also robustness and scalability in future quantum technologies.

### Measurement setup

In the realm of measurement and control instruments, digital direct synthesis technology, developed on the foundation of a radio-frequency system-on-chip architecture, has eliminated the necessity for in-phase quadrature mixers. This advancement significantly simplifies measurement circuits and enhances the integration level of measurement and control electronics. The direct generation and acquisition methods represent a primary direction for the evolution of room-temperature electronics.

In cryogenic environments, state-of-the-art dilution refrigerators now support thousands of microwave coaxial cables, enabling control over up to 1000 qubits. However, the extensive use of cryogenic circulators and high electron mobility transistors in qubit readout circuits consumes valuable low-temperature space and cooling capacity within the dilution refrigerator. Therefore, further scientific and technological advancements in qubit readout methods are essential, potentially replacing existing circuit QED schemes with more scalable solutions.

Although dilution refrigerators can achieve base temperatures as low as 10 mK, the effective temperature of quantum chips is higher due to noise, radiation and inadequate thermal anchoring. This results in a thermal population of quantum states, which degrades the fidelity of qubit reset/readout and gate operations. For multi-qubit chips, particularly those employing flip-chip technology, this issue is critical and must be addressed. A proposed solution involves a liquid helium immersion cooling scheme, as discussed in [280], but its implementation poses packaging challenges for multi-qubit chips.

Shielding against ionization caused by high-energy particles is another key concern for measurement systems. Experiments have confirmed that high-energy particles from space, such as gamma rays or muons, excite phonons in the quantum chip substrate. When the phonon energy exceeds the energy gap of the superconducting films used, Cooper pairs break, generating quasiparticles. These quasiparticles induce widespread qubit decoherence and correlated errors. To mitigate this, radiation isolation is crucial. One approach is to conduct experiments in underground facilities to minimize cosmic radiation exposure. Another method involves using gap engineering [281] techniques to reduce the harmful effects caused by quasiparticles.

### Control system

The control and optimization of scalable qubits become significantly more complex as the number of qubits increases, especially in the presence of low-frequency noise that can cause qubit parameters to drift over time. Therefore, an intelligent software control system is crucial for achieving optimal chip performance quickly. Thanks to rapid advancements in artificial intelligence (AI) technology, such systems can autonomously calibrate critical parameters like qubit frequency and coupling strength using closed-loop feedback, automatically adjust operational parameters for optimal gate fidelity with machine learning algorithms and process vast amounts of qubit data through big data analysis to extract valuable insights. Additionally, by analyzing noise affecting quantum chips and establishing corresponding models, these systems adapt to environmental changes, maintaining qubit stability and consistency. In QEC experiments, these systems achieve low-latency, high-efficiency decoding. Deeply integrating AI with multi-qubit measurement and control systems represents a pivotal direction for overcoming the limitations of manual measurements, providing new approaches for scaling larger quantum systems and paving the way for future advancements.

### Near-term applications

When will quantum computers be capable of solving real-world problems? This remains a pivotal question in the field of quantum computing. While practical, universal quantum computers are still some distance away, researchers are actively developing algorithms specifically tailored for today’s NISQ devices. These algorithms are designed to optimize the use of existing quantum resources, enabling them to address practical problems even in the presence of significant noise and a limited number of qubits. For instance, variational quantum algorithms [282] and quantum approximate optimization algorithms [283] focus on solving specific types of problems, such as chemical simulations, optimization tasks and machine learning applications. These algorithms are suitable for running on NISQ devices and are expected to find practical applications across various domains. The integration of quantum computers with high-performance computing (HPC) systems is a crucial step toward realizing the practical potential of quantum computing. This combination allows each system to leverage its strengths while compensating for the other’s weaknesses through task allocation and collaborative computing. By distributing different parts of a problem to the most appropriate computational resources, this approach facilitates real-time data exchange and collaborative work between quantum computers and HPC systems. It maximizes the parallel processing capabilities of quantum computers alongside the powerful computational resources of traditional HPC, thereby enhancing overall efficiency and performance. Moreover, interdisciplinary collaboration is becoming increasingly essential. Collaboration between physicists, computer scientists, engineers and experts from sectors such as finance and medicine fosters a better understanding of diverse industry needs, translating these needs into relevant quantum algorithms. This collaborative effort not only accelerates the development of quantum technology, but also ensures that the technologies being developed are aligned with practical requirements across various fields. In this context, quantum computing cloud platforms have played a significant role in fostering a robust ecosystem for quantum computing, enabling different industries to build upon the evolving technology. Although quantum computing has yet to achieve widespread commercial applications, the continued advancement of quantum hardware and software suggests that its potential value will gradually become apparent in the coming years. Furthermore, the increasing collaboration between industry organizations and academic institutions is accelerating this process, pushing quantum computing technology from the laboratory into real-world applications. As these advancements continue, the promise of quantum computing to solve complex, real-world problems becomes ever more tangible.

## Notes

The authors are arranged alphabetically, except for the first one. All authors contributed equally to the article.
