# Correction to: Advancements in superconducting quantum computing

**DOI:** 10.1093/nsr/nwaf582

**Published:** 2026-01-24

**Authors:** Yao-Yao Jiang, Chunqing Deng, Heng Fan, Bing-Yang Li, Luyan Sun, Xin-Sheng Tan, Weiting Wang, Guang-Ming Xue, Fei Yan, Hai-Feng Yu, Ying-Shan Zhang, Yu-Ran Zhang, Chang-Ling Zou

**Affiliations:** Beijing Key Laboratory of Fault-Tolerant Quantum Computing, Beijing Academy of Quantum Information Sciences, Beijing 100193, China; Beijing National Laboratory for Condensed Matter Physics, Institute of Physics, Chinese Academy of Sciences, Beijing 100190, China; University of Chinese Academy of Sciences, Beijing 100049, China; Quantum Science Center of Guangdong-Hong Kong-Macao Greater Bay Area, Shenzhen 518045, China; Beijing Key Laboratory of Fault-Tolerant Quantum Computing, Beijing Academy of Quantum Information Sciences, Beijing 100193, China; Beijing National Laboratory for Condensed Matter Physics, Institute of Physics, Chinese Academy of Sciences, Beijing 100190, China; Hefei National Laboratory, Hefei 230088, China; Beijing Key Laboratory of Fault-Tolerant Quantum Computing, Beijing Academy of Quantum Information Sciences, Beijing 100193, China; Beijing National Laboratory for Condensed Matter Physics, Institute of Physics, Chinese Academy of Sciences, Beijing 100190, China; University of Chinese Academy of Sciences, Beijing 100049, China; Center for Quantum Information, Institute for Interdisciplinary Information Sciences, Tsinghua University, Beijing 100084, China; Hefei National Laboratory, Hefei 230088, China; School of Physics, Nanjing University, Nanjing 210093, China; Hefei National Laboratory, Hefei 230088, China; Center for Quantum Information, Institute for Interdisciplinary Information Sciences, Tsinghua University, Beijing 100084, China; Hefei National Laboratory, Hefei 230088, China; Beijing Key Laboratory of Fault-Tolerant Quantum Computing, Beijing Academy of Quantum Information Sciences, Beijing 100193, China; Hefei National Laboratory, Hefei 230088, China; Beijing Key Laboratory of Fault-Tolerant Quantum Computing, Beijing Academy of Quantum Information Sciences, Beijing 100193, China; Beijing Key Laboratory of Fault-Tolerant Quantum Computing, Beijing Academy of Quantum Information Sciences, Beijing 100193, China; Hefei National Laboratory, Hefei 230088, China; Centre for Quantum Technologies, National University of Singapore, Singapore 117543, Singapore; School of Physics and Optoelectronics, South China University of Technology, Guangzhou 510640, China; CAS Key Laboratory of Quantum Information, University of Science and Technology of China, Hefei 230026, China; Hefei National Laboratory, Hefei 230088, China

In the article ‘Advancements in superconducting quantum computing’ (National Science Review, Volume 12, Issue 8, August 2025, nwaf246, https://doi.org/10.1093/nsr/nwaf246), a discrepancy in the author affiliations was introduced by an editorial mistake during the production process. The correct author list and affiliations are given below.

Yao-Yao Jiang^1,2,3^,*, Chunqing Deng^4^, Heng Fan^1,2,10^, Bing-Yang Li^1,2,3^, Luyan Sun^5,10^, Xin-Sheng Tan^6,10^, Weiting Wang^5,10^, Guang-Ming Xue^1,10^, Fei Yan^1^, Hai-Feng Yu^1,10^,*, Ying-Shan Zhang^7^, Yu-Ran Zhang^8^ and Chang-Ling Zou^9,10^

^1^Beijing Key Laboratory of Fault-Tolerant Quantum Computing, Beijing Academy of Quantum Information Sciences, Beijing 100193, China;

^2^Beijing National Laboratory for Condensed Matter Physics, Institute of Physics, Chinese Academy of Sciences, Beijing 100190, China;

^3^University of Chinese Academy of Sciences, Beijing 100049, China;

^4^Quantum Science Center of Guangdong-Hong Kong-Macao Greater Bay Area, Shenzhen 518045, China;

^5^Center for Quantum Information, Institute for Interdisciplinary Information Sciences, Tsinghua University, Beijing 100084, China;

^6^School of Physics, Nanjing University, Nanjing 210093, China;

^7^Centre for Quantum Technologies, National University of Singapore, Singapore 117543, Singapore;

^8^School of Physics and Optoelectronics, South China University of Technology, Guangzhou 510640, China;

^9^CAS Key Laboratory of Quantum Information, University of Science and Technology of China, Hefei 230026, China;

^10^Hefei National Laboratory, Hefei 230088, China

**Corresponding authors.** E-mails: jiangyy2022@baqis.ac.cn; hfyu@baqis.ac.cn

The authors are arranged alphabetically, except for the first one. All authors contributed equally to the article.

The list of authors in this correction is identical to that of the original article; only the affiliation information has been amended.

## Notes

The authors are arranged alphabetically, except for the first one. All authors contributed equally to the article.

